# A versatile on-axis visualization system for the macromolecular crystallography beamline BM07 at the ESRF

**DOI:** 10.1107/S1600577526010660

**Published:** 2026-10-22

**Authors:** Alexandre Appolaire, Philippe Jacquet, Lucas Petit, Eric Mathieu, Eric Girard, Sylvain Engilberge, Antoine Royant

**Affiliations:** a Univ. Grenoble Alpes, CEA, CNRS, Institut de Biologie Structurale, Grenoble, France; b European Synchrotron Radiation Facility Grenoble France; Bhabha Atomic Research Centre, India

**Keywords:** on-axis microscope, kinetic crystallography, time-resolved crystallography, high-pressure crystallography, laser excitation

## Abstract

We describe the design of a new dual-magnification on-axis microscope adapted for actinic illumination on beamline BM07 at the European Synchrotron Radiation Facility (ESRF). The instrument combines two cameras equipped with 50 mm and 150 mm focal-length imaging optics, providing low- and high-magnification views of the sample environment, respectively. In addition, the microscope incorporates two lateral optical ports for laser-light injection and collection of back-scattered fluorescence. The core of the on-axis instrument also supports motorized translations for beamstop alignment/positioning, and aperture positioning. A dedicated video and zoom server performs automatic camera selection according to the magnification requested by the user, digital zoom calculation, image-orientation correction and calibration handling. The server is integrated with the beamline software ecosystem, allowing zoom control and live video display directly through both a standalone graphical interface and the *MXCUBE* data acquisition webpage. This architecture provides a flexible platform for conventional crystal centring as well as future AI-assisted automatic sample recognition and centring, and enables light-triggered kinetic (including time-resolved) and high-pressure crystallography experiments.

## Abstract 2

A compact on-axis microscope installed on the endstation of the macromolecular crystallography beamline BM07 at the ESRF is described. The system combines dual-magnification on-axis sample visualization with on-axis laser injection and fluorescence measurements in back-scattering geometry. Its integration within the beamline control software is presented. The device is intended to support routine sample centring, light-triggered kinetic crystallography and high-pressure crystallography experiments.

## 1. Introduction

Macromolecular crystallography (MX) is currently evolving along two complementary directions. On one side, standard experiments performed under cryogenic conditions are becoming increasingly automated. Modern synchrotron beamlines now routinely offer highly integrated workflows for sample mounting, optical and X-ray centring, diffraction screening, data collection and data processing, enabling unattended data collection from large numbers of samples with no or minimal user intervention (McCarthy *et al.*, 2025 ▸; Svensson *et al.*, 2015 ▸; Wang, 2025 ▸; Sanchez-Weatherby *et al.*, 2019 ▸; Ursby *et al.*, 2020 ▸; Yamamoto & Kumasaka, 2025 ▸). On the other side, the rapid progress of AI-based structure-prediction methods, together with the increasing impact of cryo-electron microscopy, has shifted part of the role of crystallography towards questions that require experimental structural information beyond a single static model (Bourgeois & Royant, 2005 ▸; Caramello & Royant, 2024 ▸). In this context, room-temperature crystallography is experiencing a resurgence at synchrotrons (Fischer, 2021 ▸) because it provides access to structural states that can be inaccessible or distorted by cryogenic cooling. It is particularly well suited to investigate conformational heterogeneity, ligand-induced structural rearrangements, radiation-sensitive chemical states and functionally relevant motions under conditions closer to those experienced by the macromolecule during its activity (Fraser *et al.*, 2011 ▸; Becker *et al.*, 2026 ▸; Huang *et al.*, 2022 ▸, 2024 ▸; Caramello *et al.*, 2026 ▸; Stubbs *et al.*, 2026 ▸; Martel *et al.*, 2026 ▸).

This renewed interest is particularly evident in kinetic synchrotron crystallography, which includes time-resolved and temperature-resolved approaches (Caramello & Royant, 2024 ▸), where structural changes can be followed after triggering a biological reaction by light excitation. Several photoactive systems have already demonstrated the power of this approach. In bacteriorhodopsin, serial synchrotron crystallography was used to follow light-driven proton-pump intermediates (Weinert *et al.*, 2019 ▸). In other studies of microbial rhodopsins, including KR2 (Kovalev *et al.*, 2020 ▸) and xenorhodopsins (Kovalev *et al.*, 2023 ▸), optical illumination enabled the trapping and structural characterization of ion-transport states. Light-oxygen-voltage-sensing domains (LOV domains) provide another representative example. Their flavin mononucleotide chromophore forms a reversible flavin-cysteinyl photoadduct upon blue-light illumination, and this reaction has been followed by both single-crystal (Aumonier *et al.*, 2020 ▸, 2022 ▸) and serial synchrotron crystallography (Gotthard *et al.*, 2024 ▸) approaches using controlled actinic excitation. Beyond naturally photoactive proteins, optical control has also been used to drive reversible chromophore photoisomerization in fluorescent proteins (Baxter *et al.*, 2024 ▸), or to release photocaged ligands such as nitric oxide for time-resolved ligand-binding studies (Smyth *et al.*, 2025 ▸). These examples illustrate the diversity of actinic-illumination strategies now used at synchrotron beamlines, ranging from continuous or pulsed laser excitation of intrinsic chromophores to photolysis of caged compounds. More generally, they highlight the growing need for controlled optical access at the sample position when crystallography is used to probe functional states rather than only static structures.

A complementary strategy for exploring functional conformational states is to perturb the protein energy landscape by applying hydro­static pressure. High-pressure macromolecular crystallography (HPMX) using a diamond anvil cell (DAC) (Fourme *et al.*, 2009 ▸, 2011 ▸; Colloc’h *et al.*, 2006 ▸; Girard *et al.*, 2022 ▸, 2024 ▸) remains a more specialized approach than light-triggered crystallography but it provides a powerful way to shift conformational equilibria and potentially reveal high-energy structural states that can be weakly populated under ambient conditions (Girard *et al.*, 2022 ▸). Such states may be the specific target of dedicated drugs or inhibitors (Girard *et al.*, 2024 ▸). In HPMX experiments, the pressure applied to the crystal must be measured within the DAC cavity. A common approach consists of measuring the pressure-dependent shift in the fluorescence of a micrometre-sized ruby added in the DAC cavity. The ability to inject excitation light and collect fluorescence in back-scattering geometry through the on-axis microscope is therefore directly relevant to HPMX.

Together, these emerging applications place new demands on the sample-visualization systems of MX beamlines. Such systems must combine accurate optical centring with controlled light delivery, fluorescence collection and compatibility with increasingly automated workflows. On-axis viewing systems have previously been adapted to provide optical access for laser excitation and spectroscopy (Fuchs *et al.*, 2014 ▸). To meet these requirements, we have developed and installed a new dual-magnification on-axis microscope on the BM07 MX beamline at the ESRF (Caramello *et al.*, 2026 ▸). The instrument is fully compatible with conventional cryogenic MX workflows, including sample mounting with the FLEX-HCD sample changer (Papp *et al.*, 2017 ▸). The following sections describe its optical, mechanical and software architecture.

## 2. Instrument design

### 2.1. General concept

The microscope is designed as an on-axis sample-visualization system positioned along the X-ray beam direction, allowing the sample to be observed from the same direction as the incident beam. In this geometry, the optical axis of the microscope is aligned with the X-ray beam path. After calibration, the beam position is centred onto the camera image, ensuring that a sample centred in the image is also positioned at the X-ray focal point.

**Figure 1:**
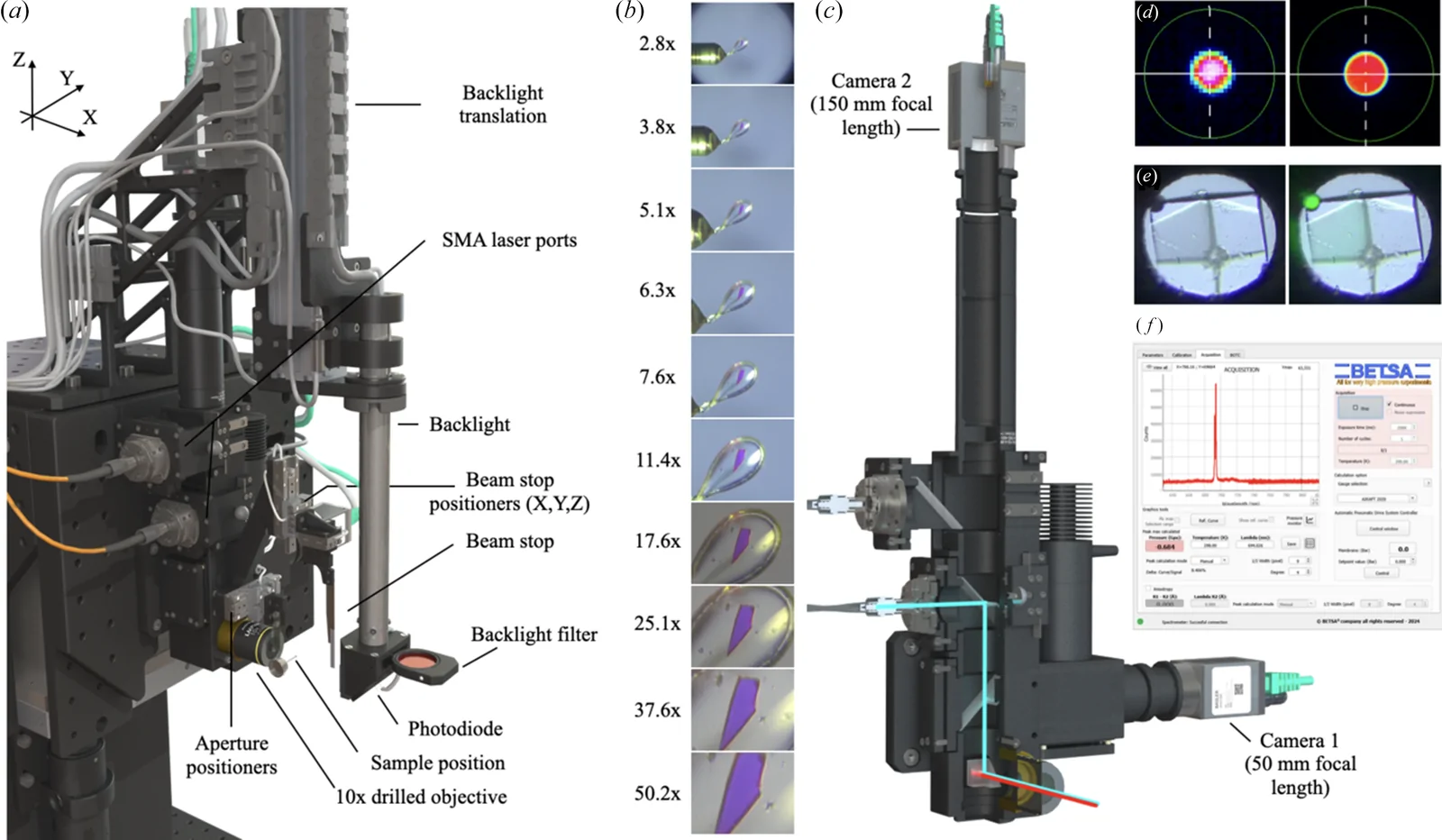
(*a*) Overview of the dual-magnification on-axis microscope installed on the sample environment of BM07. (*b*) Representative views of a mounted sample recorded at different magnification levels, from 2.8× to 50.2×, demonstrating the transition from low to high magnification. (*c*) Cutaway view of the on-axis microscope with the optical path partially exposed, showing the laser-injection path through the microscope optics (cyan beam) and the X-ray beam (red beam) passing along the optical axis through the central apertures of the prism and objective. The laser beam is redirected towards the sample by a drilled reflective prism located behind the objective; its central aperture allows passage of the X-ray beam. (*d*) Laser-beam profile 50 µm (left) and 200 µm (right) measured at the sample position using a DataRay CMOS beam profiler, illustrating the size and shape of the excitation spot delivered through the SMA-coupled optical path at the sample position. (*e*) View inside the DAC showing a lysozyme crystal in the DAC cavity (400 µm in diameter) and the 25 µm excitation laser spot at 532 nm positioned on a ruby chip (right). (*f*) Graphical user interface of the *PRL 4000* software (BETSA, France) used for ruby-fluorescence analysis, showing the fluorescence emission spectrum collected from the ruby and the corresponding pressure determination.

The base dual-magnification microscope, adapted from the OM1 model, was designed and manufactured by Suna Precision GmbH. The BM07-specific modifications used to integrate beam-conditioning elements (beamstop and apertures) around the sample position were subsequently performed in-house [Fig. 1 ▸(*a*)]. It uses a dual-magnification architecture based on 50 mm and 150 mm focal-length imaging optics, providing fields of view of 3.06 mm × 2.56 mm and 1.02 mm × 0.85 mm, respectively. Both optical paths use the same 10× Olympus (ref. LMPLFLN10X) objective with a working distance of 21 mm. This objective includes a 1.0 mm central drill hole that allows the X-ray beam to pass through the optical axis. The short focal-length provides a low-magnification/wide overview of the sample, whereas the long focal-length provides a high-magnification/narrow field of view of the crystal region. Imaging is performed using two Basler (ref. acA2440-20gc) cameras, each equipped with a 5 MP, 2/3-inch sensor and ethernet connectivity, providing a maximum of 23 frames per second (FPS) at full resolution. Regions of interest are predefined to provide smooth navigation through ten magnification levels [Fig. 1 ▸(*b*)].

Two optical ports are integrated on the side of the microscope body, enabling the connection via optical fibre to a light source (*e.g.* laser or LED) for actinic light injection and a detector for fluorescence measurements in back-scattering geometry [Fig. 1 ▸(*c*)]. The core of the on-axis microscope was modified in-house to serve as a compact support to integrate key beam-conditioning elements in the sample environment, including the beamstop and X-ray apertures. For this purpose, it is equipped with a set of SmarAct MCS2 positioners. Two SLC1720 translation stages and one SLC1750 translation stage enable beamstop positioning in the *X*, *Y* and *Z* directions. In addition, two further positioners, located above the objective, allow positioning of the X-ray apertures in the *Z* and *Y* directions using SLC1720 and SLC1750 stages, respectively [Fig. 1 ▸(*a*)]. A tungsten capillary with an inner diameter of 500 µm is inserted into the drilled objective to limit scattering from the X-ray beam.

### 2.2. Dual camera imaging

The use of two physical cameras, rather than a single camera combined with either digital or mechanical zoom (van Silfhout, 2021 ▸), offers several advantages. A mechanical zoom would require a motorized opto-mechanical assembly and result in potentially slow transitions between magnification levels. Besides, a purely digital zoom applied to a single camera would necessarily reduce the effective image resolution at high magnification. This limitation can be avoided with the dual-camera configuration: the short-focal-length camera preserves a large field of view, and quasi-instantaneous switching to the long focal one delivers the resolution required for detailed sample visualization. This is particularly important for applications such as the visualization of micrometre-sized ruby in a DAC. Magnification control, camera selection and image display are entirely software-handled, providing a continuous visualization experience either within *MXCUBE* (Oscarsson *et al.*, 2019 ▸) or through the dedicated standalone viewer developed in-house. The two cameras are managed by the control software as a single logical zoom device. For each requested zoom value, the software automatically selects the most appropriate camera and, when required, generates intermediate fields of view by applying digital zoom. The transition between both cameras thus appears seamless for the user.

### 2.3. Background illumination

The quality of background illumination is critical for reliable sample visualization and for routine alignment procedures relying on image analysis driven by artificial intelligence. For this purpose, a removable backlight system was developed and installed at the sample position. The backlight is mounted on a fast 200 mm FESTO electric translation stage (ref. ELGC-BS-KF-45-200-10P), allowing it to be inserted or retracted either by the user or automatically within predefined beamline routines. A white LED from Edmund Optics (ref. 34-217) is inserted into the core of the backlight assembly, and a 45° reflective mirror (ref. 12-454, Edmund Optics) redirects the light towards the sample. An additional diffuser (ref. 62-618, Edmund Optics) is used to scatter the light homogeneously, providing a clean and uniform background for imaging. This design provides homogeneous transmitted illumination during sample mounting, loop detection and optical centring, while allowing the backlight to be swiftly removed so as not to interfere with data collection or other optical measurements. A long-pass filter can also be inserted into the core of the backlight system, enabling safe visualization of light-sensitive samples under safe red-light conditions for instance. The design further incorporates a photodiode (ref. S11773-2, Hamamatsu) positioned behind the reflective mirror, enabling X-ray flux measurement at the sample position [Fig. 1 ▸(*a*)].

## 3. Actinic illumination for light triggered crystallographic experiments

A key feature of the microscope is the presence of two lateral optical ports (SMA type) (ref. PAF2S-11A, Thorlabs) to easily connect light sources and/or detectors via optical fibres [Figs. 1 ▸(*a*) and 1(*c*)]. The lower port incorporates a dichroic mirror with a cut-off wavelength of 552 nm (ref. 86334, Edmund Optics), and the upper port a short-pass dichroic filter with a cut-off wavelength of 650 nm (ref. 69217, Edmund Optics). Integrating these optical paths within the microscope body enables actinic illumination and/or fluorescence measurement in backscattering mode without the need for a separate optical bench and additional focusing optics around the sample position. A drilled reflective prism located immediately behind the objective redirects the optical path towards the sample, while its central aperture allows the X-ray beam to pass through along the optical axis. Note that the hole does not affect the image of the light source and does not significantly affect its intensity at the sample position. Each dichroic-mirror assembly is mounted on a mechanical drawer designed by Suna Precision GmbH, allowing straightforward insertion into or removal from the optical path without changing the apparatus settings [Figs. 1 ▸(*a*) and 1(*c*)]. The dichroic assemblies need to be removed when the system is only used for sample visualization, because they slightly alter the colour rendering of one of the imaging cameras when inserted.

The ports can be used independently or in combination: one for sample illumination and the other for collection of back-scattered fluorescence. The spot size at the sample position directly scales with fibre diameter in a 1:1 ratio, *i.e.* a 200 µm multimode fibre produces a 200 µm-diameter excitation spot [Fig. 1 ▸(*d*) and Table 1 ▸].

## 4. Ruby fluorescence measurements for HPMX

During a typical HPMX experiment, a micrometre-sized ruby is placed inside the DAC cavity, in the vicinity of the protein crystal within the sample chamber [Fig. 1 ▸(*e*)]. Upon excitation with laser light, ruby emits a characteristic fluorescence signal in the red region of the spectrum, in a narrow range centred around 694 nm, whose maximum shifts as a function of pressure and therefore provides a convenient optical pressure gauge directly inside the DAC (Mao *et al.*, 1986 ▸; Syassen, 2008 ▸; Shen *et al.*, 2020 ▸). The two dichroic mirrors described in Section 3 have been specifically selected to optimize the ruby fluorescence measurement. For HPMX experiments, the lower port will be coupled via a 25 µm multimode fibre (ref. N68L02, Thorlabs) to a 200 mW, 532 nm laser source (ref. MGL-III-532-200-3-LED-1KHZ, CNI Laser) for ruby excitation, producing a spot of ∼25 µm at the sample position [Fig. 1 ▸(*e*)] while the upper port will be connected to an HR4PRO (Ocean Optics) spectrophotometer (wavelength range: 630 to 810 nm; grating: 1200 lines mm^−1^) via a 910 µm-diameter multimode optical fibre (ref. MHP910L02, Thorlabs) to collect the ruby fluorescence in back-scattering geometry. Pressure estimation based on fluorescence measurements is performed by the *PRL 4000* software provided by BETSA [Fig. 1 ▸(*f*)].

## 5. Software architecture and beamline integration

**Figure 2:**
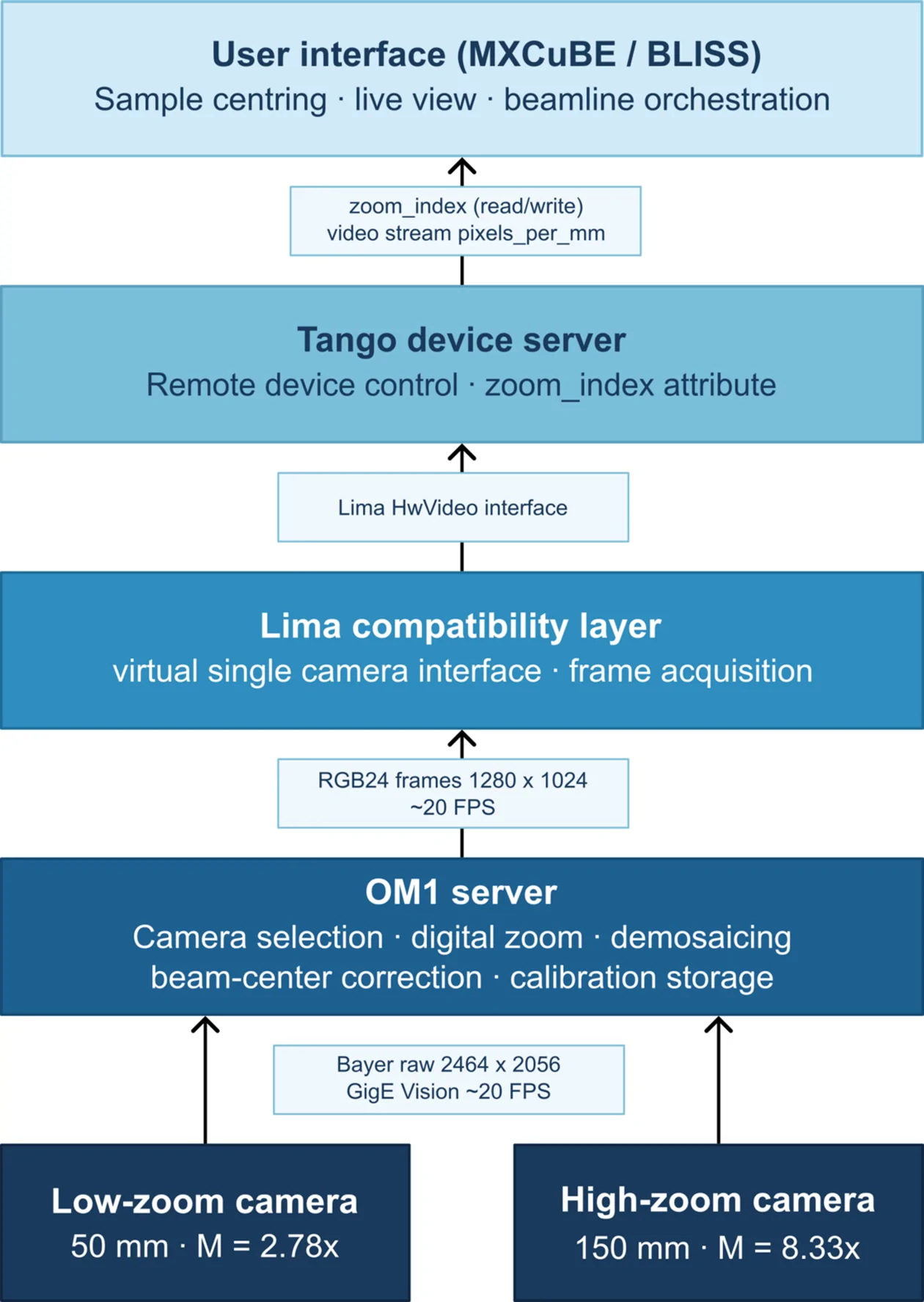
Software architecture of the on-axis dual magnification viewer system. The acquisition pipeline runs from hardware to user across five layers. The two Basler (ref. acA2440-20gc) cameras transmit raw Bayer frames with a resolution of 2464 × 2056 pixels via the GigE Vision interface to the on-axis dual magnification server (OM1 server), which performs demosaicing, beam-centre correction and digital zoom before delivering a fixed-format RGB24 stream (1280 × 1024 pixels, ∼20 FPS) to the *LIMA* layer. The *Tango* device server exposes the system as a standard network device, providing remote access to the video stream and the zoom index attribute. At the user level, *MXCUBE* is the primary client for routine operation, *i.e.* controlling zoom, displaying the live video stream and retrieving per-axis pixel-to-millimetre conversion factors for sample-centring calculations, while *BLISS* provides complementary access to the zoom index for beamline-level scripting. This architecture requires no modification to the upstream software stack, as the dual-camera system is presented to *LIMA*, *Tango* and *MXCUBE* as a single virtual on-axis viewer implementing a standard interface.

Video acquisition, streaming, zoom control and image calibration are handled by a dedicated software that acts as the central control layer of the microscope. The software stack of the on-axis dual magnification system is organized as a five-layer pipeline (Fig. 2 ▸) spanning from low-level camera acquisition to the operator interface in *MXCUBE*. The software drives both Basler cameras through the *pypylon* software development kit and performs sequential image processing on each acquired frame: colour demosaicing, a beam centring correction that aligns the optical axis with the X-ray focal point and downscaling to the fixed 1280 × 1024 pixel output. It also manages automatic camera selection according to the magnification requested by the user, calculates the digital zoom, applies orientation correction, and stores, for each magnification level, the beam-centre offset and the pixel to millimetre conversion factors required to maintain correspondence between image coordinates and actual goniometer motor positions.

Zoom control is implemented by combining hardware camera selection with software region-of-interest (ROI) cropping. The on-axis visualization system exposes ten discrete magnification levels spanning 2.8× to 50.2× [Fig. 1 ▸(*b*)]. The short focal optical path is used for low magnification (2.8× to 11.4×) while the long focal one is used for higher magnification (17.6× to 50.2×). Intermediate magnifications are produced by cropping a centred region from the full sensor frame before downscaling, so that the effective spatial sampling scales linearly with the requested zoom level. Camera switching and ROI recalculation are performed with a software latency of less than 1 ms, as determined from timestamps recorded before and after the camera-selection and image-cropping routines during both within-camera and cross-camera zoom-index transitions. This latency is therefore imperceptible to the user. In routine use, the beamline user controls the zoom directly from the *MXCUBE* interface and can monitor the live video stream in the same environment used for sample centring and data collection.

The server integrates seamlessly into the existing beamline software ecosystem by presenting the dual-camera system as a single virtual device implementing the standard on-axis viewer interface. This requires no modification to the upstream software stack composed of *LIMA* (Petitdemange *et al.*, 2018 ▸), *Tango* (Götz *et al.*, 2015 ▸), *BLISS* (Guijarro *et al.*, 2023 ▸) and *MXCUBE*, which interacts with the system exactly as they would with a conventional single-camera on-axis viewer.

This architecture has two practical advantages. First, it separates the optical hardware from the software stack, allowing future replacement or upgrade of cameras and optics without disrupting the user workflow. Second, it creates a calibrated video stream available through a standard interface that can be reused by higher-level automation tools, including automatic loop centring, crystal detection and machine-learning-based sample classification.

## 6. Future implementation

The on-axis dual-camera video stream provides a strong technical basis for AI-assisted data collection workflow. The fixed image format, calibrated pixel-to-millimetre conversion for each optical magnification, and native integration into *MXCUBE* make the system directly compatible with image-segmentation and object-detection pipelines, with limited additional preprocessing.

Automated crystal centring based on deep learning has already been demonstrated at synchrotron beamlines (Ito *et al.*, 2019 ▸). For routine sample centring, a segmentation or object-detection model should be trained to identify the loop, locate crystals, and calculate the coordinates required for automated centring. The dual-camera architecture naturally supports a two-stage workflow. The low-magnification camera would first be used to locate the sample holder, verify successful mounting on the goniometer, and provide an automated safety check during sample transfer by the FLEX-HCD sample changer (Papp *et al.*, 2017 ▸). The system would then switch to the high-magnification camera for crystal localization and fine centring.

For HPMX, similar AI models could be developed to detect and locate micrometre-scale ruby inside the DAC. This capability could be integrated into the pressure-measurement workflow, enabling automated ruby identification, positioning, and pressure measurement.

## 7. Conclusion

The dual-magnification on-axis microscope installed on the BM07 diffractometer provides a compact and versatile platform for MX workflows. By combining low- and high-magnification imaging, fibre-coupled optical access, removable back illumination, and motorized positioning of the beamstop and apertures, the system integrates several functions that would otherwise require separate devices around the sample position.

The dual-camera architecture provides an effective magnification range of 2.8× to 50.2×. The lateral SMA ports extend the microscope beyond visualization by enabling actinic illumination and induction and collection of fluorescence through the on-axis optical path. These capabilities support both light-triggered diffraction experiments and ruby-fluorescence pressure measurements for HPMX.

A dedicated server presents the instrument as a single virtual on-axis viewer within the existing *LIMA*, *Tango*, *BLISS*, and *MXCUBE* environment. This architecture enables seamless routine operation while maintaining compatibility with higher-level automation tools. The microscope therefore forms an integrated component of the BM07 endstation and provides a robust basis for future developments in automated sample recognition, high-throughput centring, room temperature kinetic and high-pressure crystallography.

## Figures and Tables

**Table 1:** Measured laser spot diameters at the sample position 2*W*_*x*_ and 2*W*_*y*_ are the spot diameters at the sample position along the *x* and *y* directions, measured at 13.5% and 50% of the peak intensity using a CMOS beam profiler from DataRay. All measured width values are given in micrometres (µm). The smallest measured spot, obtained with the 50 µm fibre, is close to the nominal minimum measurable beam diameter of the CMOS beam profiler.

Fibre diameter (µm)	2*W*_*x*_ at 13.5%	2*W*_*y*_ at 13.5%	2*W*_*x*_ at 50%	2*W*_*y*_ at 50%
50	57.0	57.3	45.1	44.8
200	207.2	207.0	191.1	191.4
400	401.2	401.2	365.5	364.5

## Data Availability

The code developed for this work is available from the corresponding author upon reasonable request.
